# The Manufacture of GMP-Grade Bone Marrow Stromal Cells with Validated In Vivo Bone-Forming Potential in an Orthopedic Clinical Center in Brazil

**DOI:** 10.1155/2019/2608482

**Published:** 2019-11-07

**Authors:** Rhayra B. Dias, João A. M. Guimarães, Marco B. Cury, Leonardo R. Rocha, Elaine S. da Costa, Liebert P. Nogueira, Camila Hochman-Mendez, Anneliese Fortuna-Costa, Anna Karoline F. Silva, Karin S. Cunha, Sergio A. L. de Souza, Maria Eugênia L. Duarte, Rafaela C. Sartore, Danielle C. Bonfim

**Affiliations:** ^1^Master Program in Musculoskeletal Sciences, National Institute of Traumatology and Orthopedics, Rio de Janeiro 20940-070, Brazil; ^2^Research Division, National Institute of Traumatology and Orthopedics, Rio de Janeiro 20940-070, Brazil; ^3^Trauma Center, National Institute of Traumatology and Orthopedics, Rio de Janeiro 20940-070, Brazil; ^4^Hip Surgery Center, National Institute of Traumatology and Orthopedics, Rio de Janeiro 20940-070, Brazil; ^5^Institute of Paediatrics and Puericulture Martagão Gesteira, Federal University of Rio de Janeiro, Rio de Janeiro 21941-912, Brazil; ^6^Institute of Clinical Dentistry, University of Oslo, Oslo 0317, Norway; ^7^Institute of Biophysics Carlos Chagas Filho, Federal University of Rio de Janeiro, Rio de Janeiro 21941-902, Brazil; ^8^Texas Heart Institute, Regenerative Medicine Research, Texas 77030, USA; ^9^Graduate Program in Pathology, Fluminense Federal University, Rio de Janeiro 24030-215, Brazil; ^10^Department of Radiology, Clementino Fraga Filho University Hospital, Federal University of Rio de Janeiro, Rio de Janeiro 21941-913, Brazil

## Abstract

In vitro-expanded bone marrow stromal cells (BMSCs) have long been proposed for the treatment of complex bone-related injuries because of their inherent potential to differentiate into multiple skeletal cell types, modulate inflammatory responses, and support angiogenesis. Although a wide variety of methods have been used to expand BMSCs on a large scale by using good manufacturing practice (GMP), little attention has been paid to whether the expansion procedures indeed allow the maintenance of critical cell characteristics and potency, which are crucial for therapeutic effectiveness. Here, we described standard procedures adopted in our facility for the manufacture of clinical-grade BMSC products with a preserved capacity to generate bone in vivo in compliance with the Brazilian regulatory guidelines for cells intended for use in humans. Bone marrow samples were obtained from trabecular bone. After cell isolation in standard monolayer flasks, BMSC expansion was subsequently performed in two cycles, in 2- and 10-layer cell factories, respectively. The average cell yield per cell factory at passage 1 was of 21.93 ± 12.81 × 10^6^ cells, while at passage 2, it was of 83.05 ± 114.72 × 10^6^ cells. All final cellular products were free from contamination with aerobic/anaerobic pathogens, mycoplasma, and bacterial endotoxins. The expanded BMSCs expressed CD73, CD90, CD105, and CD146 and were able to differentiate into osteogenic, chondrogenic, and adipogenic lineages in vitro. Most importantly, nine out of 10 of the cell products formed bone when transplanted in vivo. These validated procedures will serve as the basis for in-house BMSC manufacturing for use in clinical applications in our center.

## 1. Introduction

Bone marrow stromal cells (BMSCs) have extensively been tested at the preclinical and clinical levels for the treatment of complex bone-related injuries, such as nonunion [1–4], avascular osteonecrosis [5, 6], critical-sized defects [1, 7–12], and osteochondral lesions [13–19] because of their inherent potential to differentiate into multiple skeletal cell types [20–22], modulate inflammatory responses [23–28], and support angiogenesis [29–32].

The treatment of these conditions requires the correct combination of biological (cells and scaffolds) and mechanical factors [33–35]. To replace bone autografts—the current gold standard—in the biological component, BMSCs must be expanded in vitro on a large scale by using good manufacturing practice (GMP) [36–45]. Although a wide variety of methods have been reported to manufacture GMP-grade BMSCs, a still major challenge for the generation of BMSC products is to scale up the processes while maintaining critical cell phenotypic and functional characteristics [25, 26]. Until now, there is no consensus as to which reagents, cell culture medium, and culture systems should be used and which tests should be performed to ensure the safety and efficacy of the final product [27–29].

Therefore, for the successful translation of BMSC potential to the clinic, it is imperative to develop standard procedures for cell production, which, in addition to being evidence-based, well-documented, cost-effective, clinically practical, and incorporating GMP, also guarantee the preservation of BMSC potency [46, 47]. As one of the main orthopedic centers in Brazil, we have established an in-house facility for the isolation and large-scale expansion of functionally certified clinical-grade BMSCs. Here, we report our general procedures, which comply both with GMP standards and the Brazilian regulatory rules for cell manufacturing for therapeutic purposes. These procedures will serve as the basis for BMSC production for future applications in our center, aiming at bone repair.

## 2. Materials and Methods

### 2.1. Reagents and Materials

All reagents and materials used for BMSC isolation, expansion, and cryopreservation were certified to be of clinical grade. None of these reagents were aliquoted. To assure traceability, the lot and/or serial numbers of all reagents and materials used in each assay were registered. Reagents used in in vitro differentiation assays and immunophenotyping, when not of clinical grade, were certified to have been produced under good laboratory practices and had defined chemical purity standards. A complete list of all reagents used with all related information is available in Supplementary Materials (Supplementary [Supplementary-material supplementary-material-1]). The technical procedures described herein were adapted to comply with the rules of the Brazilian Health Regulatory Agency (ANVISA, Collegiate Board Resolution—RDC Nos. 09/2011 and 214/2018) for the development of cell products intended for use in humans.

### 2.2. Sample Donor Eligibility Criteria

The study design, the procedures used for its execution, and sample collection were approved by the institutional ethics committee (No. 41660415.3.0000.5273). Subjects of both sexes who were older than 18 years old with indications for total hip replacement were invited to donate the discarded acetabular bone/marrow. After providing written informed consent to donate the samples, a brief questionnaire was used to assess the risk of blood-borne infections. The donors were also questioned about previously diagnosed comorbidities, such as rheumatoid arthritis, diabetes mellitus, bone marrow dysplasia, and malignant tumors, the use of immunosuppressant drugs, and any history of drug and/or alcohol abuse. If any of these were disclosed, the donor was excluded from the study. To those selected, further inclusion criteria were applied: a platelet count > 150 × 10^9^/L, a hemoglobin concentration > 11.5 g/dL for women and 12.5 g/dL for men (the reference values for blood donation—ANVISA, RDC No. 153/2004), and a negative pregnancy test. Donor blood samples were also tested for HIV-1/2, hepatitis B virus surface antigen, hepatitis B virus core antigen, hepatitis C virus, HTLV-1, HTLV-2, cytomegalovirus, *Mycobacterium tuberculosis*, *Treponema pallidum*, and *Trypanosoma cruzi* infection. In the case of any positive test, the donor was excluded from the study. The data for the final selected donors is summarized in Table 1.

### 2.3. Preparation of Total Bone Marrow Cell Suspensions

Acetabular bone fragments were processed immediately after harvesting or after a maximum of 12 h of storage at 2-8°C in *α*-minimum essential medium (*α*-MEM, LGC Biotechnology, São Paulo, SP, BRA) supplemented with 20% fetal bovine serum (FBS, Gibco-Thermo Fisher, Waltham, MA, USA) [37, 48–50]. The samples were added to 50 mL tubes, and phosphate-buffered saline (PBS, Amresco, Solon, OH, USA) was added at a 1 : 4 (*w*/*v*) ratio. After vigorous mechanical homogenization with a 10 mL pipette (the speed of the hand pipettor must be set to high), bone spicules were allowed to settle, and the cell suspension was collected and transferred to a new 50 mL tube. Fresh PBS was added to the tube containing the spicules, at the same ratio, and a second round of homogenization was performed. After brief spicule sedimentation, the supernatant was collected and transferred to a new tube. This step was repeated at least three times or until the bones were visually clean of marrow. The collected marrow suspensions were centrifuged at 300 × *g* for 5 minutes. After centrifugation, a 5 mL sample of the supernatant was collected for bacterial contamination testing. The pelleted cells were resuspended in *α*-MEM supplemented with 20% FBS. To determine the number of total nucleated cells obtained, an aliquot of the marrow suspension was diluted in an appropriate amount of 2% glacial acetic acid for red blood cell lysis and subsequently loaded in a modified Neubauer chamber for cell counting.

### 2.4. Colony-Forming Efficiency (CFE) Assay

To estimate the number of colony-forming units (CFU-Fs) in the obtained marrow suspensions, nucleated cells were plated in triplicate at a density of 8, 0 × 10^3^/cm^2^ (Corning Incorporated, New York, NY, USA) in 2 mL of *α*-MEM supplemented with 20% FBS. After 3 days of incubation at 37°C in 5% CO_2_, the nonadherent hematopoietic cells were removed, and the medium was changed. At day 14, the colonies were fixed with 4% paraformaldehyde (Sigma-Aldrich, St. Louis, MO, USA) and stained with 1% crystal violet (Sigma-Aldrich). Colonies with more than 50 cells were counted [48]. The efficiency of colony formation was expressed as the mean colony number relative to the 100000 bone marrow nucleated cells plated.

### 2.5. BMSC Isolation

To determine the optimal cell seeding density for BMSC isolation, nucleated cells from samples 01 to 04 were resuspended at 0.08 × 10^5^/cm^2^, 0.4 × 10^5^/cm^2^, and 2.0 × 10^5^/cm^2^ in 10 mL of *α*-MEM supplemented with 20% FBS and plated in triplicate in T-75 cm^2^ flasks (Corning Incorporated). After plating, the cells were allowed to adhere for 3 days in a humidified atmosphere of 5% CO_2_ at 37°C. Then, the nonadherent cells were removed, the adherent cells were washed three times with PBS, and the medium was changed. Thereafter, the adherent cells were allowed to proliferate for 11 additional days. Complete medium exchange was performed every 3 days. At day 14, a 5 mL aliquot of the culture medium was collected for bacterial contamination testing. The cells were washed twice with PBS and harvested with recombinant trypsin (TrypLE® Express, Invitrogen, Carlsbad, CA, USA). The cell number was determined by manual counting with a Neubauer chamber. The cell viability was assessed by the Trypan Blue exclusion method. If the cell viability was <70%, the cells were discarded and expansion was stopped. For samples 05 to 14, nucleated cells were plated only at 0.4 × 10^5^/cm^2^, and the isolation was performed as described.

### 2.6. Large-Scale BMSC Expansion

Large-scale BMSC expansion was performed in multilayer cell factories, in a two-step process [37, 45]. In the first expansion cycle (passage 1—P1), BMSCs were seeded in 2-layer cell factories (1264 cm^2^, Corning Incorporated) at a density of 2.0 × 10^3^ cells/cm^2^ in 500 mL of *α*-MEM supplemented with 20% FBS. The cells were incubated at 37°C in 5% CO_2_ and allowed to proliferate until the monolayers reached 70% confluence. The medium was changed every 3 days. To estimate the degree of confluence of the cells in the cell factories, sentinel T-75 cm^2^ flasks were simultaneously seeded with BMSCs and kept under the same culture conditions [37]. During harvesting, the cells were washed two times with PBS and incubated for 10 minutes with 150 mL of TrypLE® Express to induce detachment. After centrifugation at 300 × *g* for 5 minutes, the cells were resuspended in fresh expansion medium and counted in a Neubauer chamber as described above. Expansion was discontinued if the cell viability was <70%.

For the second expansion cycle, BMSCs were seeded in 10-layer cell factories (6320 cm^2^, Corning Incorporated) at a density of 2.0 × 10^3^ cells/cm^2^ in 1.5 L of *α*-MEM supplemented with 20% FBS. When the cells reached 70% confluence, a 5 mL aliquot of the culture medium was collected for bacterial contamination testing, and the cells were washed twice with PBS. After 10 minutes of incubation with TrypLE® Express, the cell suspension was collected, diluted 1 : 2 (*v*/*v*) in Ringer's lactate solution (Fresenius Kabi, Bad Homburg vor der Höhe, GER) supplemented with 5% human albumin (Alburex® 20, CSL Behring AGB, Berna, SWE), and centrifuged at 300 × *g* for 5 minutes. The cell pellets were then washed five times with Ringer's lactate solution supplemented with 0.5% human albumin to remove the FBS proteins [37]. Finally, the cells were resuspended in Ringer's lactate solution with 5% human albumin and counted in a Neubauer chamber. The cell viability was assessed with Trypan Blue staining. If the cell viability was >70%, the cells were either used in subsequent experiments or processed for cryopreservation.

### 2.7. Population Doubling Analysis

In each expansion cycle, the number of population doublings (PD) was calculated using the formula PD = (Log *N*_f_‐Log *N*_i_)/Log 2, in which *N*_f_ is the final harvested cell number and *N*_i_ is the initial seeded cell number. The cumulative PD (cPD) was calculated by adding PD_1_ and PD_2_. The doubling time (dT) was determined by dividing the time in days required for total cell expansion by the cumulative PD (dT = Δ*t*/cPD) [51].

### 2.8. Cryopreservation and Storage

A total of 5.0 × 10^6^ BMSCs were resuspended in 1.0 mL of cryopreservation solution consisting of 5% dimethylsulfoxide (DMSO, Sigma-Aldrich), 5% human serum albumin, and 6% hydroxyethyl starch solution (Voluven®, Fresenius Kabi) in Ringer's lactate solution. The cryotubes were placed in a room temperature Mr. Frosty freezing container (Nalgene®, Sigma-Aldrich), which was immediately transferred to a -80°C freezer. After overnight incubation, the vials were transferred to boxes that were stored in the vapor phase of a liquid nitrogen tank.

### 2.9. Viability of the Cryopreserved Cells

After four and 40 weeks of cryopreservation in the aforementioned conditions, one vial from five BMSC products (07–11) was thawed in a water bath at 37°C. The cell suspension was immediately diluted 1 : 10 (*v*/*v*) in *α*-MEM supplemented with 20% FBS and centrifuged at 300 × *g* for 5 minutes. The supernatants were discarded, and the cells were resuspended in 4 mL *α*-MEM supplemented with 20% FBS. The number of dead and live cells was determined by manual counting with a Neubauer chamber, using the Trypan Blue exclusion method.

### 2.10. Bacterial, Endotoxin, and Mycoplasma Contamination Testing

To evaluate the sterility of the final BMSC products, the presence of aerobic and anaerobic bacteria and mycoplasma and the levels of endotoxin were evaluated. For aerobic and anaerobic bacterial contamination testing, 1 mL of the cell culture media collected at the end of bone marrow cell suspension preparations and at the end of P0 (isolation) and P2 was inoculated into BD BACTEC™ Plus Aerobic/F and BD BACTEC™ Plus Anaerobic/F (Becton Dickenson, New Jersey, USA) culture vials and incubated for 10 days.

Mycoplasma contamination and endotoxin levels were evaluated in P2 culture media with the detection kits MycoAlert™ and LAL Pyrogent™-5000 (both from Lonza, Basel, SWI), respectively. Measurements were performed in accordance with the manufacturer's protocol. Values ≤ 1.2 for mycoplasma and 5 UE/mL for endotoxin (reference values) were considered negative.

### 2.11. Quantification of Bovine Transferrin

Levels of FBS proteins in the final cell products were estimated by the quantification of bovine transferrin concentration by ELISA. After the cells' washing step with Ringer's lactate, a 2 mL sample of the cell suspension was collected and measurements were performed according to the manufacturer's instructions (Abnova, Taipei, TWN). Values ≤ 10 ng/mL (assay reference value) were considered negative.

### 2.12. Immunophenotyping

For immunophenotypic characterization of cells, 1.0 × 10^6^ BMSCs per tube were washed with FACS buffer consisting of PBS supplemented with 1% bovine serum albumin (BSA, Sigma). Then, the cells were incubated for 30 minutes in the dark at room temperature with the following fluorochrome-conjugated primary antibodies: anti-CD90-Percp-Cy5.5, anti-CD73-APC, anti-CD105-FITC, anti-CD146-PE (all from BioLegend, San Diego, CA, USA), anti-CD14-FITC (Immunostep, Salamanca, SPA), anti-CD34-FITC, anti-CD45-Percp-Cy5.5 (both from Agilent DAKO, Santa Clara, CA, USA), and anti-CD11b-PE (Santa Cruz Biotechnology, Dallas, TX, USA). The isotype controls were IgG2A-FITC, IgG1A-APC, IgG1A-Percp-Cy5.5, IgG1-PE, IgG1-FITC, and IgG2A-PE (all from Santa Cruz Biotechnology). Next, the cells were washed with FACS buffer and resuspended in 300 *μ*L buffer for acquisition with a BD FACSCanto™ cytometer (BD Biosciences). The data were analyzed with FlowJo software (Tree Star, Ashland, OR, USA).

### 2.13. In Vitro Osteogenic Differentiation and Von Kossa Staining

BMSCs were plated at a density of 1.3 × 10^4^ cells/cm^2^ in triplicate in *α*-MEM supplemented with 20% FBS and allowed to grow until 100% confluence was reached. Osteogenic differentiation was induced by incubation with *α*-MEM containing 10 mM *β*-glycerophosphate, 5 *μ*g/mL ascorbic acid 2-phosphate, and 10^−6^ M dexamethasone (all from Sigma) supplemented with 20% FBS for 21 days, and the medium was changed every 3 days [51]. To assess the mineralization, monolayers were stained with Von Kossa. The cells were fixed in 4% paraformaldehyde for 10 minutes at room temperature and incubated with 2% silver nitrate (Sigma) aqueous solution for 40 minutes while protected from light. The cells were washed three times with distilled water and exposed to UV light for 10 minutes. The wells were photographed using an Eclipse TS100 inverted microscope (Nikon, Tokyo, JPN).

### 2.14. In Vitro Adipogenic Differentiation and Oil Red O Staining

BMSCs were plated as described above. After reaching 100% confluence, the cells were incubated with *α*-MEM containing 0.5 mM isobutylmethylxanthine, 200 mM indomethacin, 10^−6^ M dexamethasone (all from Sigma), and 10 mM insulin (Humulin®, Lilly, São Paulo, SP, BRA) supplemented with 20% FBS for 21 days, and the medium was changed every 3 days [51]. To confirm the lipidic composition of the cell vacuoles, BMSCs were fixed in 4% paraformaldehyde for 10 minutes at room temperature, washed with propylene glycol PA (Vetec Quimica, Rio de Janeiro, RJ, BRA), and incubated with 0.5% Oil Red O solution (Sigma) in propylene glycol for 20 minutes. After two washes with 85% propylene glycol, the cells were photographed using a Nikon Eclipse TS100 inverted microscope.

### 2.15. In Vitro Chondrogenic Differentiation

BMSCs were resuspended in *α*-MEM supplemented with 20% FBS at a density of 1.0 × 10^7^ cells/mL. One 10 *μ*L drop of this cell suspension was placed in each well in a U-shaped 96-well plate, which was incubated at 37°C in 5% CO_2_ for 30 minutes. Then, 100 *μ*L of StemPro® chondrogenic medium (Thermo Fischer) was added to each well. The medium was changed every 2 days for 21 days [50, 52–55]. The formed micromasses were fixed in 4% paraformaldehyde for 3 h at room temperature, embedded in paraffin, cut into 5 *μ*m sections, and stained with H&E or Masson's Trichrome stain (EasyPath, São Paulo, SP, BRA) according to the manufacturer's instructions. The slides were photographed using a Nikon E600 microscope.

### 2.16. Type II Collagen Immunofluorescence

To confirm type II collagen deposition in the chondrogenically induced micromasses, 5 *μ*m paraffin sections were obtained as described above and incubated in citrate buffer (pH 6.0) at 96°C for 40 minutes. After blocking with 10% BSA for 1 h, the sections were incubated overnight at 4°C with an anti-collagen type II primary antibody (sc-288887, Santa Cruz Biotechnology) diluted 1 : 50 in Tris-buffered saline (TBS) with 1% BSA. Subsequently, the slides were washed three times with TBS and incubated for 2 h with an Alexa Fluor 546-conjugated secondary antibody (Life Technologies, Thermo Fisher) in the dark at room temperature. The nuclei were stained with 1 *μ*g/mL DAPI solution (sc-3598, Santa Cruz Biotechnology). Fluorescence images were obtained using a Leica TCS SP5 laser scanning confocal microscope (Leica Microsystems, Wetzlar, GER).

### 2.17. Subcutaneous Xenotransplantation Assay

To evaluate the in vivo bone-forming potential of the expanded BMSCs, 1.0 × 10^6^ cells were mixed with 30 mg of hydroxyapatite/tricalcium phosphate powder (HA/TCP, Osteoset® T, Wright Medical, Arlington, TN, USA) in 1 mL of *α*-MEM supplemented with 20% FBS in a 1.5 mL tube. The cell/HA/TCP mixture was incubated overnight at 37°C to allow sedimentation and cell attachment to the HA/TCP particles. Then, the supernatant was carefully aspirated, and 15 *μ*L of 3.2 mg/mL human fibrinogen and 100 U/mL human thrombin (both from Sigma) were added to form a fibrin glue [55–58]. After 3 h of incubation, the cell/HA/TCP implant was collected and subcutaneously transplanted into the flank of an immunocompromised mouse (beige BALB/c nu/nu, IPEN, São Paulo, SP, BR) aged between 6 and 8 weeks [50, 55]. For each BMSC sample, three implants were transplanted per mouse: one cell-free (negative control) and two containing the cells; and two mice were used. Surgeries were performed under general anesthesia with intraperitoneal injections of 80-100 mg/g ketamine hydrochloride and 10 mg/kg xylazine. After 12 weeks, the mice were euthanized by deep anesthesia, and the implants were harvested. All animal procedures were performed in accordance with the guidelines of the Institutional Animal Care and Use Committee (002/2014).

### 2.18. Implant Histology and Immunohistochemistry

Following harvesting, the implants were fixed for 24 h in 4% paraformaldehyde in PBS. For routine histology, the implants were decalcified by incubation in 10% nitric acid (Vetec) for 3 days, processed for paraffin embedding, cut into 6 *μ*m sections, and stained with H&E.

For immunohistochemistry, the implants were decalcified in 10% EDTA (Sigma) for 8-12 weeks and processed for paraffin embedding and sectioning as previously described. The sections were incubated overnight at 4°C with rabbit anti-lamin A/C antibody (M00438, Boster, Pleasanton, CA, USA) diluted 1 : 100 or with mouse anti-collagen type I antibody (Abcam, Cambridge, UK) diluted 1 : 300. After 2 washes with EnVision™ FLEX Wash Buffer (DAKO Agilent, Santa Clara, CA, USA), the sections were incubated for 2 h with EnVision Flex (DAKO Agilent). The signal was developed in EnVision Flex Substrate Buffer containing 20 *μ*L/mL DAB—EnVision Flex DAB+ Chromogen (DAKO Agilent)—for 3 min. All images of the glass slides were obtained by digital scanning with an Aperio CS2 scanner and ImageScope software (both from Leica Biosystems).

### 2.19. Assessment of Bone Density by Micro-CT

Micro-CT scans were performed using the desktop SkyScan1172 scanner (Bruker, Brussels, BEL). One implant from each sample was placed in customized tubes and wrapped in gauze dampened with 4% formalin to avoid shrinkage. The scanning parameters were as follows: 4.0 *μ*m isotropic pixel size and an X-ray source with 70 kV accelerating voltage and 141 *μ*A current with a 500 *μ*m Al filter. The samples were rotated 180° around their vertical axis with a rotational step of 0.4°, an exposure time of 1770 ms, and frame averaging of 3. The total scan time per sample was approximately 1.2 h. The images were reconstructed with NRecon v.1.7.1.0 software (Bruker) using a filtered back-projection algorithm. The 3D images were rendered using CTVox software (Bruker). To discriminate bone from HA/TCP, the grayscale images were binarized. For binarization, the threshold value was chosen based on a visual comparison of the original grayscale image and the binary image for each tissue slice. The binary samples were quantified in CTAn software (Bruker).

### 2.20. Statistical Analysis

The values were expressed either individually and/or as the mean/median, as appropriate. The Gaussian distribution was evaluated by the Shapiro-Wilk and Kolmogorov-Smirnov tests. Comparisons between groups were evaluated either by one-way ANOVA with Tukey's multiple comparisons test or by Kruskal Wallis with Dunn's multiple comparisons test. *P* values ≤ 0.05 were considered significant. Analyses were performed with GraphPad Prism software (GraphPad software version 8.0, La Jolla, CA, USA).

## 3. Results

### 3.1. Patient and Sample Characteristics

Trabecular bone containing marrow was collected from nine females and five males with ages ranging between 48 and 75 years old (Table 1). The frequency of clonogenic cells in the samples was estimated by the colony-forming efficiency (CFE) assay. The average number of colonies harvested from each sample was heterogeneous and ranged from 7.66 to 48.0 CFU-Fs per 100000 nucleated cells (mean of 25.29 ± 13.20 colonies). The size of the colonies also varied, with average diameters ranging from 1.8 mm to 9.1 mm (mean of 4.5 ± 1.86 mm) (Table 1).

### 3.2. Optimization of Cell Seeding Density for BMSC Isolation

**Figure 1:**
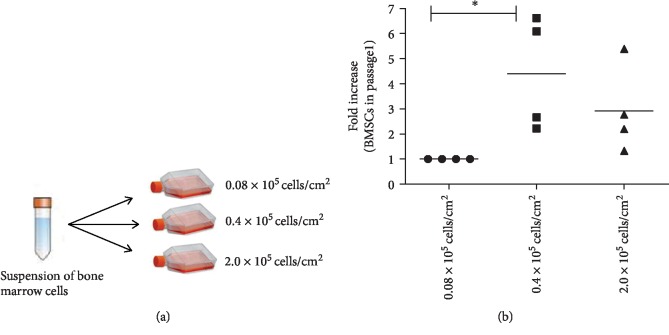
Standardization of cell isolation. (a) Schematic representation of the protocol used for determining the optimal initial seeding densities. (b) Fold increase in cell number (BMSCs in passage 1). *n* = 4; *p* = 0.015.

To determine the optimal conditions for improved BMSC isolation from the bone marrow total nucleated cell fractions, BMSC samples 01–04 were seeded at three cell densities: 0.08 × 10^5^/cm^2^, which was the density used in the CFE assays, 0.4 × 10^5^/cm^2^, and 2.0 × 10^5^/cm^2^ (Figure 1(a)). The density of 0.4 × 10^5^ cells/cm^2^ yielded a significantly higher number of BMSCs in comparison with the clonal density of 0.08 × 10^5^/cm^2^ (Figure 1(b)) and, therefore, was used as the standard for BMSC isolation in subsequent samples.

### 3.3. BMSC Manufacturing

**Figure 2:**
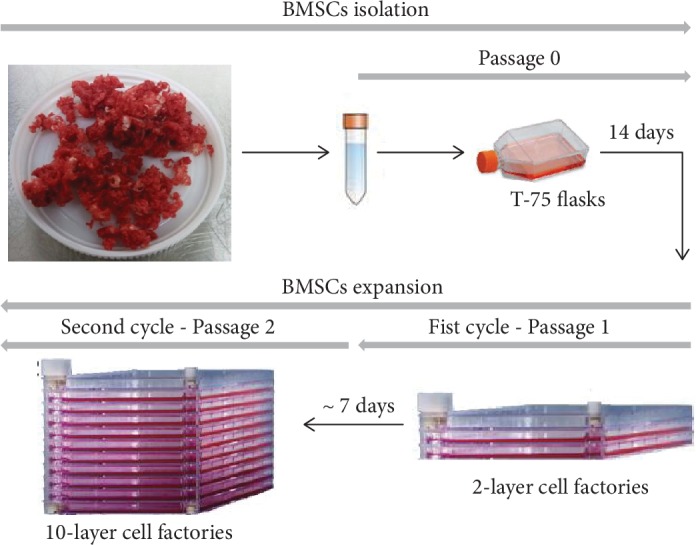
Schematic representation of the in vitro bone marrow stromal cells manufacturing in a GMP-compliant semiclosed system. After bone marrow dissociation from bone spicules, nucleated cells were seeded in T-75 flasks for BMSC isolation (P0), followed by expansion in 2-layer cell factories (P1) and subsequent expansion in 10-layer cell factories (P2).

BMSC manufacturing was performed in three steps: isolation in T-75 flasks (P0), followed by two rounds of expansion in 2- and 10-layer cell factories (Figure 2). The average BMSC harvest per T-75 flask at the end of P0 was 0.49 ± 0.20 × 10^6^ cells (Table 2). The minimal BMSC number required to initiate expansion in a 2-layer cell factory was obtained for all 10 bone marrow samples. Average viability was of 95.67% ± 4.33%.

In P1, the cells reached 70% confluence in 5.8 ± 1.75 days. The average BMSC yield per 2-layer cell factory was 21.93 ± 12.81 × 10^6^ cells, and the average viability was 95.29% ± 3.51% (Table 2). Once again, all samples reached the minimum cell number required for subculture. In P2, the cells reached confluence after 6.6 ± 0.69 days, and an average of 83.05 ± 114.72 × 10^5^ cells were harvested per 10-layer cell factory. Viability was of 93.53% ± 5.14%. Considering both cycles of expansion, the total time needed to obtain the final cell product was 26.4 ± 2.33 days (Table 2).

### 3.4. Analysis of Population Doubling

With the adopted procedures for BMSC manufacturing, cells doubled an average of 10.74 ± 2.60 times during P0, 2.93 ± 0.75 times during P1, and 2.29 ± 1.18 during P2 (Table 3). The time for population doubling was of 1.18 days during P0 and 2.37 days during P1, reaching 3.10 days during P2, which was significantly increased in comparison to P0 (Table 3).

### 3.5. Sterility Analysis of BMSC Products

To attest the sterility of bone samples and BMSCs and ultimately the quality of the technical procedures and the whole facility environment, tests for aerobic and anaerobic pathogens were performed at P0 and P2 in cell supernatants. No bacterial growth was ever detected (Table 4). The tests for mycoplasma and pyrogenic substances were also all negative (Table 4).

### 3.6. Assessment of Animal Protein Content in Final BMSC Products

At the end of expansion, BMSCs were washed five times with Ringer's lactate solution containing 0.5% human albumin. Then, the levels of residual animal-derived proteins in the final cell products were evaluated by the quantification of bovine transferrin. None of the samples showed a level above the limiting value of 10 ng/mL (Table 4), indicating that the BMSC products were compliant with GMP conditions.

### 3.7. Immunophenotypic Characterization and In Vitro Differentiation of Expanded BMSCs

**Figure 3:**
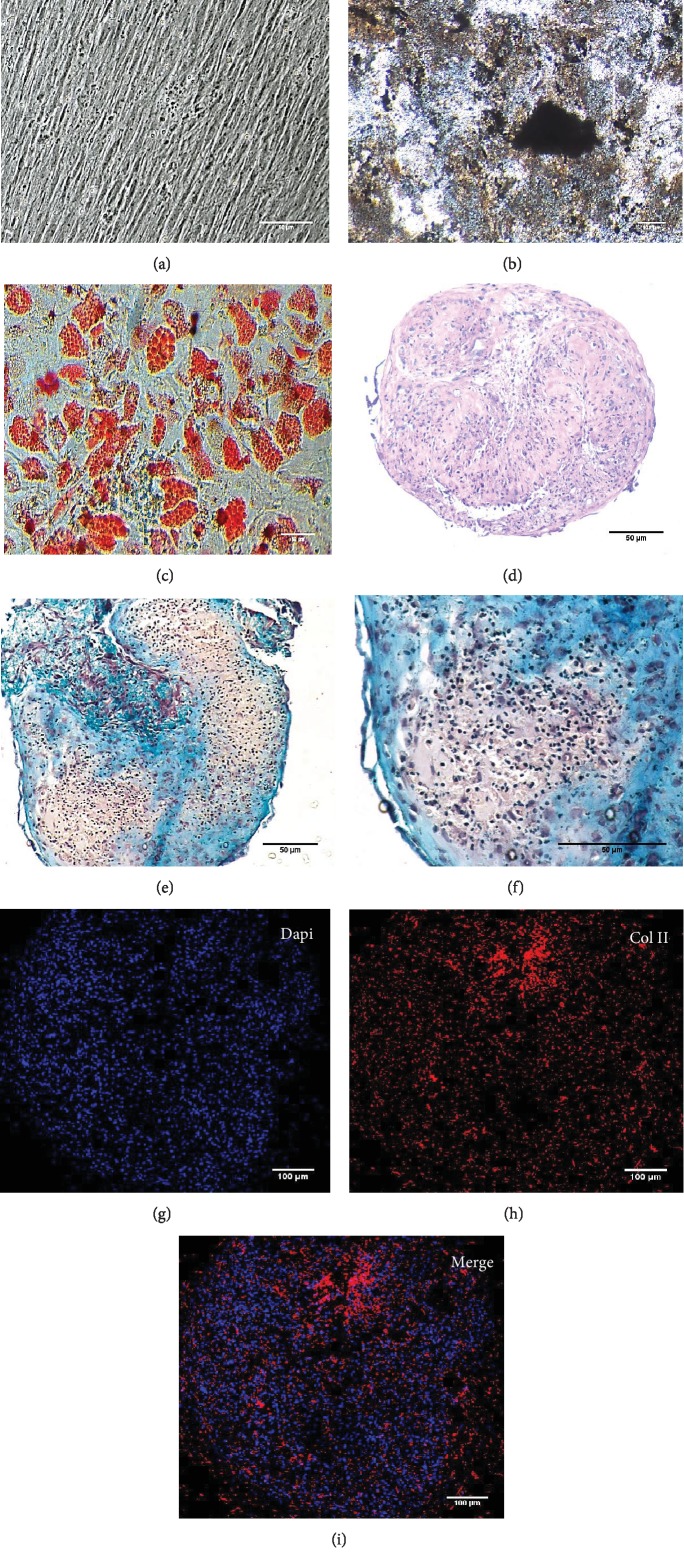
In vitro osteogenic, adipogenic, and chondrogenic potential of BMSC products. Representative images of (a) a noninduced BMSC layer, (b) mineralized nodules visualized by Von Kossa staining, and (c) intracellular lipid accumulation stained with Oil Red O. (d–i) Chondrogenic differentiation: (d) H&E staining, (e, f) Masson Trichrome staining, and (g-i) immunofluorescence staining of collagen II in representative BMSC micromass pellet cultures with differentiation towards the chondrogenic lineage. Cartilage matrix deposition (blue) in the extracellular matrix was assessed by Masson Trichrome staining and confirmed by immunofluorescence staining for collagen II. Representative images of *n* = 10 experiments. For the immunofluorescence analysis, *n* = 3.

To characterize the cell products, we first assessed the expression of BMSC-related cell surface markers by FACS. The expanded BMSCs homogenously expressed CD73, CD90, CD105, and CD146 and were negative for the hematopoietic lineage markers CD34, CD45, CD14, and CD11b (Table 5 and Supplementary [Supplementary-material supplementary-material-1]). Next, we evaluated their differentiation potential in vitro. The induced BMSCs were able to differentiate into osteoblasts, adipocytes, and chondrocytes (Figure 3 and Supplementary [Supplementary-material supplementary-material-1]), as shown by the deposition of mineralized nodules with positive Von Kossa staining (Figure 3(b)), intracellular lipid accumulation with positive Oil Red O staining (Figure 3(c)), and collagen type II-rich cartilaginous matrix as revealed by Masson's Trichrome stain (Figures 3(e) and 3(f)) and immunofluorescence analysis (Figures 3(g)–3(i)).

### 3.8. Assessment of the In Vivo Bone-Forming Potential

To verify whether the BMSCs in the final cell products indeed retained the ability to differentiate and form bone in vivo, the cells were subcutaneously transplanted into immunodeficient mice. The histological examination of the implants revealed the formation of ossicles for nine out of 10 BMSC products (Table 6).

**Figure 4:**
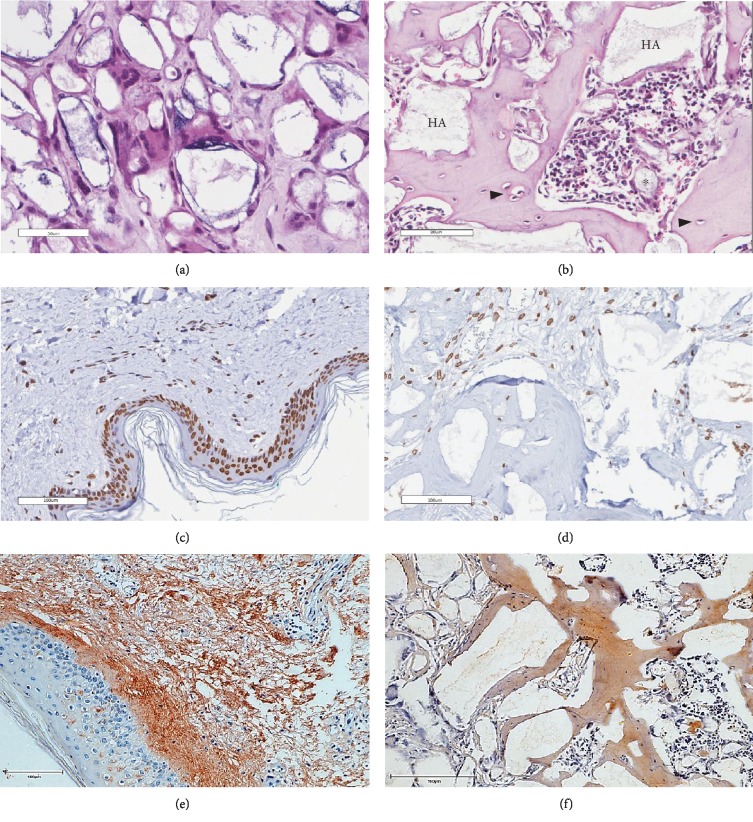
Osteogenic potential of BMSCs in vivo. In vivo transplantation assays were performed by combining BMSCs with HA/TCP followed by subcutaneous transplantation into immunocompromised mice. (a, b) H&E staining. (a) Negative control (HA/TCP transplantation without BMSC). (b) BMSCs formed ectopic ossicles that were sometimes populated by host hematopoietic marrow (asterisk). The arrowheads indicate osteocytes. HA = hydroxyapatite/tricalcium phosphate particles. (c–f) The human origin of the woven bone by immunohistochemical analysis. (c, e) Positive control of lamin A/C and collagen I stains, respectively, in human skin. (d, f) Expression of human lamin A/C and collagen I within the woven bone (for the immunohistochemistry analysis, *n* = 3).

The bone matrix was deposited over the surfaces of the HA/TCP particles and resembled trabecular bone architecture (Figures 4(b), 4(d), and 4(f)). Osteocytes were embedded in the newly synthesized matrix (Figure 4(b), arrowheads), and the reconstituted marrow stroma was filled with hematopoietic cells (Figure 4(b), asterisk). The human origin of the cells inside the ossicles was corroborated through human lamin A/C and type I collagen detection within the woven bone (Figures 4(d) and 4(f) and Supplementary [Supplementary-material supplementary-material-1]). Micro-CT-based 3D reconstruction of the BMSC implants (Supplementary [Supplementary-material supplementary-material-1]) showed that the neoformed bone density ranged from 994 to 1946 HU (Hounsfield unit), which was in accordance with the reference density values described for human cancellous and cortical bone (Table 6).

### 3.9. Analysis of Cryopreservation Conditions

Finally, to evaluate the quality of the cryopreservation procedures, vials of five BMSC lots were thawed after four and 40 weeks of storage. Cell viability was similar at both time points and did not significantly differ from the percent viability at the time of cryopreservation (Table 7).

## 4. Discussion

Because of their osteogenic, immunomodulatory, and angiogenesis-promoting potential, BMSCs have been the focus of extensive research of their clinical application in orthopedics [59]. However, the translation to the bedside still faces important bottlenecks, one being the lack of regulatory and technical consensus determining the overall conditions that should be adopted for BMSC manufacturing and what assays should be performed in order to validate the cell potency [25, 26]. In this sense, centers in different countries have adopted its own procedures, resulting in the manufacturing of BMSC products with distinct gene expression signatures and functional potentials. In Brazil, the sanitary regulatory health agency (ANVISA) provided general regulatory rules for the establishment of facilities for cell manufacturing and/or processing for therapeutic purposes but left to the facilities the decision about what technical procedures should be adopted for primary tissue harvesting, processing, cell isolation, expansion, and characterization, according to the particularities of the cells to be manufactured. In this report, we described our procedures to generate functionally validated BMSC products that conform to both GMP standards and national regulatory policies.

In order to have a parameter for comparison, our protocols were based on the previous experience of the NIH BMSC bank [37]. But differently from their protocol, in which cells were expanded in three cycles (one in 2-layer and two in 10-layer cell factories), in our manufacturing process, cells were expanded in only two subsequent rounds in 2- and 10-layer cell factories, respectively. Another difference was the source of bone marrow. Instead of using bone marrow aspirates from volunteered donors, we used trabecular bone discards. The main reason for this choice was the fact that at this initial point our goal was simply to set and evaluate the procedures for BMSC manufacturing in our in-house facility. Because we are housed in an orthopedic hospital, bone discards from primary total hip arthroplasties (THA) are always available and can be obtained without subjecting donors to any additional procedures. And from previous studies of our group using BMSCs isolated from THA as a model, the capacity of these cells to proliferate and differentiate in vitro and in vivo was already known [50], thus proving its usefulness to validate our procedures.

Indeed, the number of clonogenic cells in the bone marrow of the THA samples, as assessed by the CFE assay, was within the previously reported range of CFE measured in bone specimens from eight healthy adults [48] and was, on average, three times higher than the CFE of bone marrow aspirates reported in the NIH BMSC bank manuscript [37]. This was not surprising because marrow aspirates are usually more contaminated with peripheral blood, which dilutes the marrow, decreasing CFE counts. Also, BMSCs were successfully isolated from all 14 samples.

Although the time for population doubling progressively increased during expansion—which can be a reflex of the donors' age—the total average cell yield obtained using just one cell factory per cycle (our minimal infrastructure capacity) showed that a significant number of cells can be produced with our protocol, considering a lot unit of 100 × 10^6^ BMSCs as proposed by the NIH facility [37]. Maintaining proliferation limited to 15–20 population doublings (including both the isolation and the two expansion cycles), cell output can be increased just by scaling up the number of T-75 flasks and cell factories accordingly to sample availability, the cell yield at each passage, and the maximum infrastructure capacity of the facility. Noteworthily, we did not use the whole amount of THA bone discards for cell isolation, as we did not aim to produce the highest number of cells as possible, but rather to determine the average yield of cells at each step.

Because ANVISA and international regulatory organizations, such as the US Food and Drug Administration (FDA), have recommended that reagents from animal sources should be avoided when manufacturing cells for human use because of the risk of zoonoses and xenogeneic immunological reactions, efforts have been made in the field to identify substitutes for fetal bovine serum, such as chemically defined media [60–63], human serum [64], and activated platelet lysates [65–68]. Although platelet lysates were shown to induce BMSC proliferation as efficiently as FBS [43, 66], strong evidence that these BMSC products have similar functional potency as FBS-expanded BMSCs has not been provided so far. Indeed, Ren and colleagues [65] showed that the substitution of FBS with platelet lysates during BMSC isolation and expansion resulted in BMSCs with different genes and microRNA expression profiles. While FBS-cultured BMSCs expressed genes involved in the MAPK, TGF-*β*, adhesion, and extracellular matrix pathways, BMSCs cultured with platelet lysates expressed genes related to metabolic, proliferation, cell cycle, and immune response pathways. Functional assays with either of these BMSC products were not performed [65]. Therefore, in line with the standard protocol used by the NIH cell bank [37], we included FBS in the BMSC isolation and expansion media but added washing steps for the final cell products to reduce the animal protein content. The BMSC lots were shown to have animal protein concentrations below the threshold limit stipulated by the FDA, indicating that the cell products had no increased risks of infection that would hamper their use in patients.

Finally, because at present no known phenotypic or genetic characteristic allows the prospective determination of the differentiation ability of a given population of BMSCs once transplanted in vivo [69, 70] and in vitro assays are believed to have a high probability of being artifactual [49, 71–73], we established the in vivo differentiation assay as the critical test to ascertain BMSC potency. Although the in vivo assay takes months to complete and to obtain a result, it is long recognized by scientists of the field as the best available method to evaluate the intrinsic differentiation potential of a given cell population, as it allows the inherent expression of cell potency without any exogenous chemical inducer [21, 49, 58, 74, 75]. By using this functional quality control method, we showed that nine out of 10 of our BMSC products formed bone in vivo with the expected histology and matrix microarchitecture. Additionally, of the nine samples that formed bone, four developed a supportive hematopoietic stroma, which is an indicator of the presence of multipotential skeletal stem cells in a BMSC population [20, 49, 70, 76]. Therefore, we concluded that the protocol we established for large-scale BMSC manufacturing preserved the potential of cells to form bone in vivo, and we confirmed its suitability for the future generation of cells for bone repair strategies. This protocol can be equally used for BMSC isolation from any source sample. The only adaptation needed if the source sample is a bone marrow aspirate is the adjustment of the initial total nucleated cell seeding density for BMSC isolation, which needs to be higher because of the decreased CFE counts of the aspirates. For this adaptation, we refer the reader to the report of the NIH BMSC bank [37].

Although we have shown that BMSCs isolated from THA bone discards can form bone in vivo, which suggests that these samples could be useful for BMSC banking for allogeneic use, we emphasize that the choice for autologous or allogeneic cell applications, as well as the decision of the best bone marrow source material, needs to be carefully evaluated on a case by case basis. Because cell application itself was beyond our present objective, we did not conduct immunogenicity and/or HLA compatibility tests, but in any allogenic strategy scenario, these tests should be conducted, in addition to the tests presented herein.

## 5. Conclusion

Due to the implementation of regulatory rules for the establishment of facilities for cell manufacturing by the Brazilian sanitary agency, a significant step towards the clinical use of BMSCs in orthopedics was taken. However, definition of the procedures for the large-scale production of clinical-grade BMSCs with validated bone-forming potential in vivo is still needed. In this study, we described a protocol for the generation of BMSC products with certified phenotype and capacity to form bone after in vivo transplantation, which will serve as the basis for in-house BMSC manufacturing for future clinical applications in our center.

## Figures and Tables

**Table 1:** Characteristics of donor patients and colony-forming efficiency (CFE) results.

	Gender	Age	TNC (10^5^)	CFU-F/10^5^	Colony diameter (mm)
BMSC 01	M	54	620	7.66	9.1
BMSC 02	F	61	520	8.33	8.4
BMSC 03	F	54	600	19.33	5.0
BMSC 04	M	48	590	17.33	4.8
BMSC 05	F	55	350	13.5	4.0
BMSC 06	M	65	150	45.33	3.9
BMSC 07	F	75	150	23.66	4.3
BMSC 08	F	75	150	20.33	2.5
BMSC 09	F	64	280	45	4.8
BMSC 10	M	71	250	48	1.8
BMSC 11	M	57	370	31	3.8
BMSC 12	F	54	210	20.33	3.3
BMSC 13	F	74	540	23	4.0
BMSC 14	F	70	250	31.33	3.3
Mean ± SD	—	62.0 ± 9.30	359.28 ± 173.55	25.29 ± 13.20	4.5 ± 1.86

TNC: total nucleated cells; CFU-F: colony-forming unit-fibroblast.

**Table 2:** Yield (number) and viability of BMSCs at each culture step.

	BMSC isolation (P0)			Passage 1 (P1)				Passage 2 (P2)				
	Cells seeded (10^6^)/T-75	Yield (10^6^)/T-75	%viability	Cells seeded (10^6^)	Yield (10^6^)	%viability	Time (days)	Cells seeded (10^6^)	Yield (10^6^)	%viability	Time (days)	Total production time (days)
BMSC 05	3.0	0.35	100	2.5	42.0	95	6	12	118.0	95	7	27
BMSC 06	3.0	0.92	90.5	2.5	30.0	98.1	3	12	56.0	95.2	6	23
BMSC 07	3.0	0.72	100	2.5	43.7	100	3	12	34.0	100	6	23
BMSC 08	3.0	0.52	92.3	2.5	12.0	96	4	12	40.0	98.7	5	23
BMSC 09	3.0	0.62	87.9	2.5	27.0	92.8	7	12	400.0	85.6	7	28
BMSC 10	3.0	0.37	100	2.5	12.0	90	7	12	26.0	92	7	28
BMSC 11	3.0	0.32	96.7	2.5	12.1	90.2	7	12	60.0	89.5	7	28
BMSC 12	3.0	0.41	97.3	2.5	15.0	98	7	12	20.0	97.5	7	28
BMSC 13	3.0	0.37	93.7	2.5	12.5	98.7	7	12	40.0	96.1	7	28
BMSC 14	3.0	0.31	98.3	2.5	13.0	94.1	7	12	36.5	85.7	7	28
Mean ± SD	**—**	0.49 ± 0.20	95.67 ± 4.33	**—**	21.93 ± 12.81	95.29 ± 3.51	5.8 ± 1.75	**—**	83.05 ± 114.72	93.53 ± 5.14	6.6 ± 0.69	26.4 ± 2.33

**Table 3:** Number of population doublings and the doubling time for each BMSC passage.

	Population doubling				Time for population doubling (days)			
	P0	P1	P2	Cpd		P0	P1	P2
BMSC 05	9.75	4.07	3.30	17.12		1.43	1.47	2.12
BMSC 06	3.23	3.58	2.22	9.04		4.33	0.84	2.70
BMSC 07	12.31	4.13	1.50	17.94		1.13	0.73	3.99
BMSC 08	12.05	2.26	2.00	16.31		1.16	1.77	2.50
BMSC 09	11.85	3.43	5.06	20.34		1.18	2.04	1.38
BMSC 10	11.02	2.26	1.50	15.51		1.26	3.09	4.66
BMSC 11	11.74	2.28	2.58	16.60		1.19	3.08	2.71
BMSC 12	11.99	2.58	0.74	15.31		1.16	2.71	9.50
BMSC 13	12.08	2.32	2.00	16.04		1.15	3.01	3.50
BMSC 14	11.37	2.38	1.99	15.74		1.23	2.94	3.52
Mean ± SD	10.74 ± 2.60	2.93 ± 0.75	2.29 ± 1.18	15.99 ± 2.85	*Median*	**1.18**	**2.37**	**3.10** ^∗^

Cpd: cumulative population doubling. ^∗^*P* = 0,004 vs. P0; Kruskal Wallis with Dunn's multiple comparisons test.

**Table 4:** Purity and safety analysis of the BMSCs.

	Anaerobic and aerobic bacteria^∗^	Mycoplasma<1.2^∗∗^	Endotoxin < 5 EU/mL^∗∗^	Bovine transferrin < 10 ng/mL^∗∗∗^
BMSC 05	ND	0.57	<0.005	7.43
BMSC 06	ND	0.43	<0.005	0.04
BMSC 07	ND	0.33	<0.005	0.04
BMSC 08	ND	0.42	<0.005	0.03
BMSC 09	ND	1.08	<0.005	0.04
BMSC 10	ND	0.82	<0.005	0.05
BMSC 11	ND	0.37	<0.005	ND
BMSC 12	ND	0.47	<0.005	ND
BMSC 13	ND	0.63	<0.022	ND
BMSC 14	ND	0.70	<0.005	ND

ND: not detected. ^∗^Assessed in the cell supernatant during sample collection, BMSC isolation, and expansion (passages 0 and 2). ^∗∗^Assessed in the supernatant from passage 2 BMSCs. ^∗∗∗^Assessed in the supernatant from passage 2 BMSCs washed for 5 cycles with Ringer's lactate solution containing 0.5% human albumin to reduce the residual level of bovine contaminants.

**Table 5:** Immunophenotypic characterization of the lots of BMSCs that were produced.

	%CD73	%CD90	%CD105	%CD146	%CD73/CD90/CD105/CD146	%CD11b	%CD14	%CD34	%CD45
BMSC 05	99.8	99.8	62.3	99.7	70.2	0.59	2.20	1.34	3.21
BMSC 06	100	100	82.3	99.9	82.3	0.86	1.85	0.56	2.85
BMSC 07	100	100	83.3	99.1	73.4	2.38	0.69	0.16	3.32
BMSC 08	97.6	97.6	100	100	95	0.25	0.11	0.45	0.98
BMSC 09	100	98.1	66.4	98.3	50.1	0.69	1.24	0.55	2.39
BMSC 10	92.7	99.6	99.4	99.3	98.8	0.62	0.18	0.22	2.85
BMSC 11	99.7	97.1	95.8	88.4	62.9	1.35	1.14	2.17	2.67
BMSC 12	99.9	99.4	75.2	100	86.1	1.03	0.11	0.11	1.26
BMSC 13	100	100	89.2	100	72.2	3.56	0.19	0.80	4.18
BMSC 14	99.7	98.9	98.3	99.1	82.9	0.12	0.08	1.09	0.26
Mean ± SD	98.94 ± 2.19	99.06 ± 1.02	85.24 ± 13.09	98.38 ± 3.36	77.39 ± 13.95	1.14 ± 1.00	0.77 ± 0.74	0.74 ± 0.60	2.39 ± 1.14

**Table 6:** Osteogenic potential of the lots of BMSCs produced.

	Tissues formed	Bone density (HU)^∗^
BMSC 05	Bone/bone marrow	1572
BMSC 06	Bone	1233
BMSC 07	Bone/bone marrow	994
BMSC 08	Bone	1209
BMSC 09	Bone	1605
BMSC 10	Bone/bone marrow	1363
BMSC 11	Bone	1946
BMSC 12	Bone	1074
BMSC 13	Bone/bone marrow	1356
BMSC 14	Fibrous tissue	—

^∗^Reference values: 700 HU (cancellous bone); 3000 HU (cortical bone).

**Table 7:** Viability evaluation of the cryopreserved lots of BMSCs.

	At the time of cryopreservation (%)	After 4 weeks of cryopreservation (%)	After 40 weeks of cryopreservation (%)
BMSC 07	100	78	85
BMSC 08	98.7	92	84
BMSC 09	85.6	93	95
BMSC 10	92	91	75
BMSC 11	89.5	88	78
Mean ± SD	93.16 ± 6.11	88.4 ± 6.10	83.4 ± 7.70

*P* = 0.11 (one-way ANOVA with Tukey's multiple comparisons test).

## Data Availability

All data used to support the findings of this study are included within the article and the supplementary information file.

## Supplementary Materials

Supplementary Materials Supplementary Figure 1: immunophenotypic characterization of BMSC products. Figure a shows representative flow cytometry histograms used to obtain the percentage of cells expressing the surface marker in question. In b, it is shown the gating strategy for the simultaneous analysis of CD73, CD90, CD105, and CD146 surface markers. Supplementary Figure 2: quantifications of the mineralized nodules positive for Von Kossa stain, the number of vacuoles with intracellular lipid accumulation positive for Oil Red O, and cartilaginous matrix positive for Masson's Trichrome stain. Supplementary Figure 3: negative controls of the immunohistochemistry analysis showing no recognition of mouse collagen type I and lamin A/C by the primary antibodies designed to recognize the given human proteins. Supplementary Figure 4: reconstruction of bone implants by micro-CT. The images show the contrast generated in the micro-CT images in function of the density, the thickness, and the energy of the X-rays, which were used to discriminate the new bone and the HA/TCP scaffold. Supplementary Table 1: list of all reagents and materials used.
