# Supplementary material for: The Manufacture of GMP-Grade Bone Marrow Stromal Cells with Validated In Vivo Bone-Forming Potential in an Orthopedic Clinical Center in Brazil
Source: Stem Cells Int. 2019 Nov 7;2019:2608482. doi: 10.1155/2019/2608482 (PMC6875385; doi:10.1155/2019/2608482)
Supplement: Supplementary Materials — Supplementary Figure 1: immunophenotypic characterization of BMSC products. Figure a shows representative flow cytometry histograms used to obtain the percentage of cells expressing the surface marker in question. In b, it is shown the gating strategy for the simultaneous analysis of CD73, CD90, CD105, and CD146 surface markers. Supplementary Figure 2: quantifications of the mineralized nodules positive for Von Kossa stain, the number of vacuoles with intracellular lipid accumulation positive for Oil Red O, and cartilaginous matrix positive for Masson's Trichrome stain. Supplementary Figure 3: negative controls of the immunohistochemistry analysis showing no recognition of mouse collagen type I and lamin A/C by the primary antibodies designed to recognize the given human proteins. Supplementary Figure 4: reconstruction of bone implants by micro-CT. The images show the contrast generated in the micro-CT images in function of the density, the thickness, and the energy of the X-rays, which were used to discriminate the new bone and the HA/TCP scaffold. Supplementary Table 1: list of all reagents and materials used. [file 2608482.f1.pdf]

The manufacture of GMP-grade bone marrow stromal cells with validated in vivo bone-forming potential in an orthopedic clinical center in Brazil

Rhayra B. Dias[1,2], João A. M. Guimarães[2,3], Marco B. Cury[4], Leonardo R. Rocha[2,3], Elaine S. da Costa[5], Liebert P. Nogueira[6], Camila Hochman-Mendez[7,8], Anneliese Fortuna-Costa[2], Anna Karoline F. Silva[9], Karin S. Cunha[9], Sergio A. L. de Souza[10], Maria Eugênia L. Duarte[2], Rafaela C. Sartore[2], Danielle C. Bonfim[2].

Supplementary Material

Supplementary Figure 1: Immunophenotypic characterization of BMSC products. Figure “a” shows representative flow cytometry histograms used to obtain the percentage of cells expressing the surface marker in question. In “b”, it is shown the gating strategy for the simultaneous analysis of CD73, CD90, CD105, and CD146 surface markers.

Supplementary Figure 2: Quantifications of the mineralized nodules positive for Von Kossa stain, the number of vacuoles with intracellular lipid accumulation positive for Oil Red O, and cartilaginous matrix positive for Masson’s Trichrome stain.

Supplementary Figure 3: Negative controls of the immunohistochemistry analysis showing no recognition of mouse collagen Type I and Lamin A/C by the primary antibodies designed to recognize the given human proteins.

Supplementary Figure 4: Reconstruction of bone implants by micro-CT. The images showing the contrast generated in the microCT images in function of the density, the thickness and the energy of the x-rays, that were used to discriminate the new bone and the HA/TCP scaffold.

Supplementary Table 1: List of all reagents and materials used.

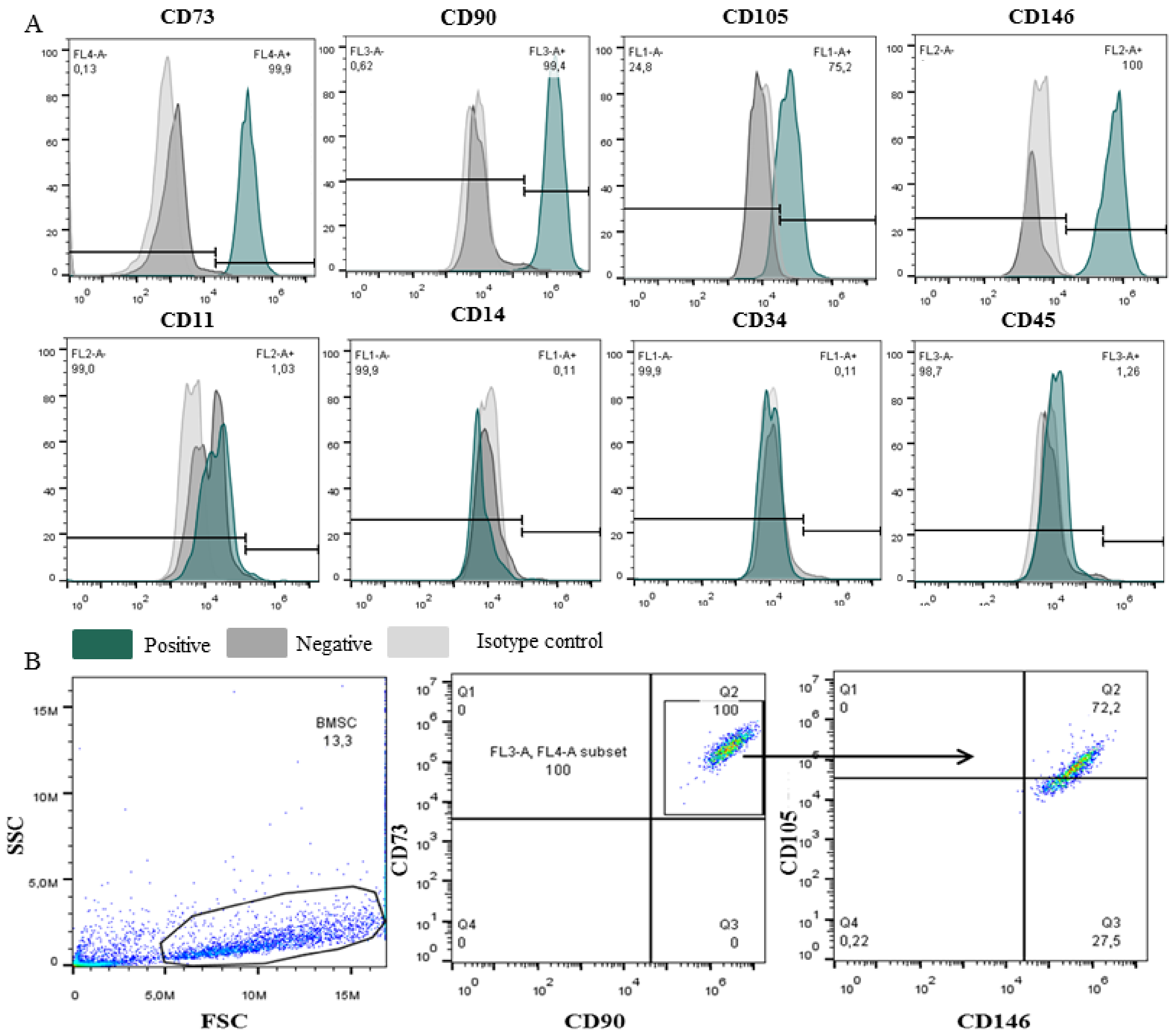

Supplementary Figure 1. Immunophenotypic profile of the BMSCs. (a) Representative flow cytometry histograms (from the BMSC 14 sample) showing the percentages of cells expressing CD90, CD73, CD105, CD146, CD11b, CD14, CD34 and CD45 (green) compared to the corresponding isotypes (dark gray) and unlabeled cells (light gray). (b) Representative flow cytometry plots showing the percentages of cells simultaneously expressing CD73, CD90, CD105 and CD146 (from the BMSC 15 sample).

To quantify the extent of mineralization and the number of fat accumulating cells, wells were photographed using an inverted microscope (Nikon Eclipse TS100). The mineralized area in 10-15 random fields was quantified using the NIH Image J software (NIH, Maryland, USA) and was represented as the percentage of the total area. The number of fat accumulating cells was expressed as the number of cells with intracellular lipid accumulation per field of view in 10 random fields. In the chondrogenic differentiation, images of 2-3 histological sections containing the whole micromass area were acquired with a 4X objective using the RGB-mode illumination on an EVOS M5000 microscope (Thermofischer scientific). The Fiji plugin Trainable Weka Segmentation (NIH software) was used for the quantitative measurement of the cartilaginous area according to the Masson’s Trichrome stain.

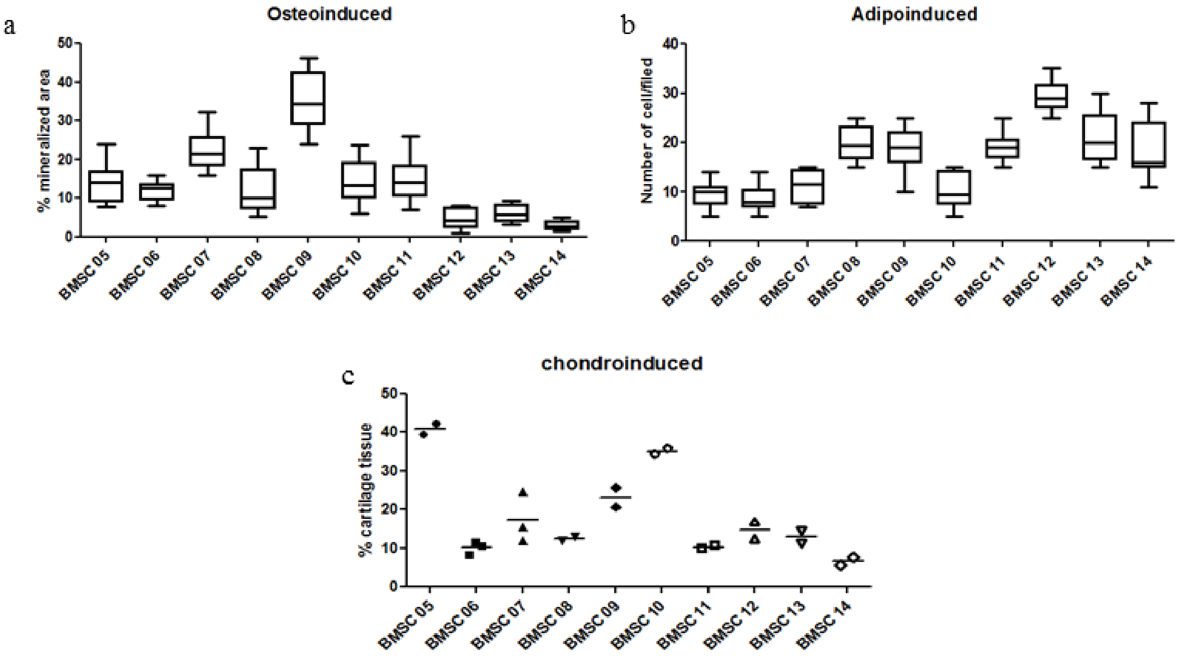

Supplementary Figure 2. Quantification of the extent of in vitro osteogenic, adipogenic, and chondrogenic differentiation. (a) Percent of the mineralized area positive for Von Kossa stain. (b) Number of cells with intracellular lipid accumulation positive for Oil Red O. (c) Percent of cartilaginous area positive for Masson’s Trichrome stain.

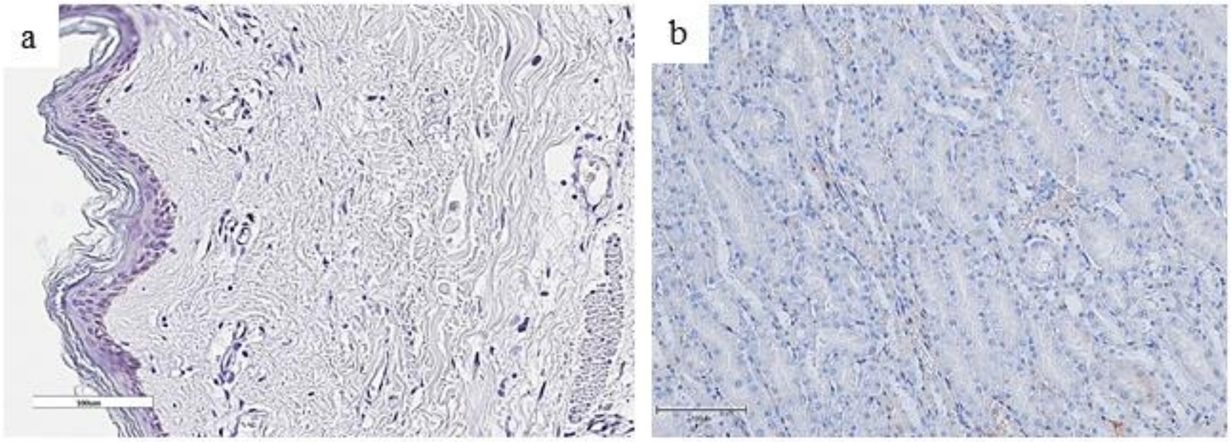

Supplementary Figure 3. Immunohistochemical analysis of collagen I and lamin A/C. Negative controls showing no staining with the primary antibodies against human collagen I in mouse skin (a) and human lamin A/C in mouse kidney (b).

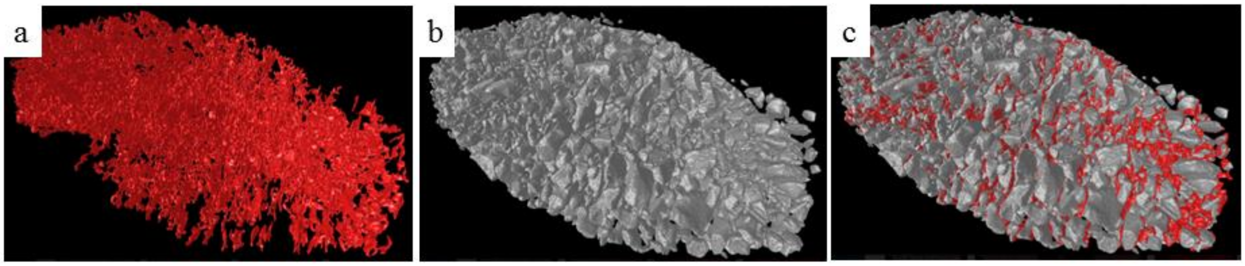

Supplementary Figure 4. 3D reconstruction of micro-CT images. red = new bone; gray = HA/TCP scaffold. Representative images of n = 9 experiments.

Table S1. List of reagents and materials

Reagent	Source	Identifier	Related information
2-layer cell factories	Corning Incorporated	CLS3310	-
10-layer cell factories	Corning Incorporated	CLS3312	-
T-75 flask	Corning Incorporated	CLS3275	-
Mr. Frosty freezing container	Sigma-Aldrich	C1562	-
3-Isobutyl-1-methylxanthine	Sigma-Aldrich	I7018	BioUltra, ≥99%*1
Alexa Fluor 546 secondary antibody	Invitrogen - Thermo Fisher	A11010	-
Anti-lamin A/C	Bosterbio	M00438	-
Anti-collagen type II	Santa Cruz	sc-288887	-
Anti-collagen type I	Abcam	ab90395	-
IgG2A-FITC	Santa Cruz	SC-2856	-
IgG1A-APC	Santa Cruz	SC-2888	-
IgG1A-Percp-Cy5.5	Santa Cruz	SC-45123	-
IgG1-PE	Santa Cruz	SC-2877	-
IgG1-FITC	Santa Cruz	SC-2855	-
IgG2A-PE	Santa Cruz	SC-2867	-
Anti-CD90-Percp-Cy5.5	Biolegend	328118	-
Anti-CD73-APC	Biolegend	560847	-
Anti-CD105-FITC	Biolegend	120405	-
Anti-CD146-PE	Biolegend	342004	-
Anti-CD14-FITC	Immunostep	EP-12-15173	-
Anti-CD34-FITC	Dako Agilent	PNIM1870U	-
Anti-CD45-Percp-Cy5.5	Dako Agilent	PNIM2653U	-
Anti-CD11b-PE	Santa Cruz	SC-65429	-

Ascorbic acid 2-phosphate	Sigma-Aldrich	795437	ACS[*2]
BACTEC plus aerobic	BD	442023	IVD[*3]<br><br>ANVISA registration MS10033430658
BACTEC plus anaerobic	BD	442022	IVD<br><br>ANVISA registration MS10033430658
Bovine serum albumin	Sigma-Aldrich	A1470	BioReagent, ≥98%[*4]
Bovine transferrin kit	Abnova	A1470	-
DAPI	Santa Cruz	sc-3598	-
Dexamethasone	Sigma-Aldrich	D9184	USP[*5]
Dimethylsulfoxide	Sigma-Aldrich	D2650	BioReagent, ≥99.7%<br><br>Sterile and tested for endotoxin levels
EDTA	Sigma-Aldrich	E4884	ACS
EnVision FLEX Systems	Dako Agilent	K800221-2	IVD
Fetal bovine serum	Gibco	26140079	Qualified[*6]
Fibrinogen	Sigma-Aldrich	F3879	-
Glacial acetic acid	Sigma-Aldrich	1005706	USP
Human albumin	CSL Behring	102568ah	Clinical grade<br><br>ANVISA registration MS 1.0151.0122
Insulin	Lilly	D.C.B. 04918	Clinical grade<br><br>ANVISA registration MS 1.1260.0057
Hydroxyethyl starch solution	Fresenius Kabi	1400391FFX-30	Clinical grade<br><br>ANVISA registration M.S. 1.0041.0099
Indomethacin	Sigma-Aldrich	I8280	USP
Masson’s Trichrome	EasyPath	EP-11-20013	IVD

MycoAlert™ Kit	Lonza	LT07-118	-
Nitric acid	VETEC	V800282	ACS
Oil Red O	Sigma-Aldrich	O0625	Certified by the Biological Stain Commission
Osteoset®	Wright Medical	130764-4	Clinical grade
Paraformaldehyde	Sigma-Aldrich	P6148	Reagent grade
PBS	Amresco	VWRV0780	Ultra Pure grade
Pyrogent™-5000	Lonza	N383	IVD<br>ANVISA registration MS 80670780002
Propylene glycol	Sigma-Aldrich	P4347	USP
Recombinant trypsin	Gibco-Thermo	12605010	Animal Origin-Free[*7]
Ringer's lactate	Fresenius Kabi	PR228F2	Clinical grade<br>M.S. 1.0041.0103
Silver nitrate	Sigma-Aldrich	209139	ACS
Sodium citrate	Sigma-Aldrich	1613859	USP
StemPro® chondrogenic medium	Gibco	A1007101	RUO[*8]
Human Thrombin	Sigma-Aldrich	T9326	BioUltra, ≥95%
α-MEM	LGC Biotechnology	BR30007-05	Endotoxin level: < 10 EU/m and Mycoplasma Negative
β-glycerophosphate	Sigma-Aldrich	G9422	BioUltra, ≥ 99%

*1: According to Sigma-Aldrich, BioUltra reagents are the "finest quality products for life science, with the most stringent testing protocols and highest purity. Superior manufacturing methods and product specific testing protocols are designed to ensure quality to our highest standard. Tests may include, but are not limited to, purity by gel electrophoresis, trace metal testing and application testing".

*2: ACS reagents meets or exceeds purity standards set by the American Chemical Society (ACS). This grade is acceptable for food, drug, or medicinal use and can be used for ACS applications or for general procedures that require stringent quality specifications and a purity of ≥95%

*3: IVD, in vitro diagnostic use

*4: Base grade products, suitability tested for specific applications such as molecular biology or cell culture.

*5: USP grade meets or exceeds requirements of the United States Pharmacopeia (USP). This grade is acceptable for food, drug, or medicinal use.

*6: ISO 13485 certified, processed in FDA registered facilities. Labeled for in-vitro diagnostics (compliant with highest level of USP sterility testing) Endotoxin level: < 10 EU/mL, Hemoglobin level: < 25 mg/dL

*7: Manufacturing is compliant with cGMP requirements

*8: Research use only. Regularly tested for endotoxin levels and sterility, according to the USP. Endotoxin Level: ≤ 50 EU/ml and serum Free
